# Atomic Force Microscopy Characterization of Protein Fibrils Formed by the Amyloidogenic Region of the Bacterial Protein MinE on Mica and a Supported Lipid Bilayer

**DOI:** 10.1371/journal.pone.0142506

**Published:** 2015-11-12

**Authors:** Ya-Ling Chiang, Yuan-Chih Chang, I-Chen Chiang, Huey-Ming Mak, Ing-Shouh Hwang, Yu-Ling Shih

**Affiliations:** 1 Institute of Physics, Academia Sinica, Taipei, Taiwan; 2 Department of Materials Science and Engineering, National Tsing Hua University, Hsinchu, Taiwan; 3 Institute of Cellular and Organismic Biology, Academia Sinica, Taipei, Taiwan; 4 Institute of Biological Chemistry, Academia Sinica, Taipei, Taiwan; 5 Institute of Biochemical Sciences, National Taiwan University, Taipei, Taiwan; University of Waterloo, CANADA

## Abstract

Amyloid fibrils play a crucial role in many human diseases and are found to function in a range of physiological processes from bacteria to human. They have also been gaining importance in nanotechnology applications. Understanding the mechanisms behind amyloid formation can help develop strategies towards the prevention of fibrillation processes or create new technological applications. It is thus essential to observe the structures of amyloids and their self-assembly processes at the nanometer-scale resolution under physiological conditions. In this work, we used highly force-sensitive frequency-modulation atomic force microscopy (FM-AFM) to characterize the fibril structures formed by the N-terminal domain of a bacterial division protein MinE in solution. The approach enables us to investigate the fibril morphology and protofibril organization over time progression and in response to changes in ionic strength, molecular crowding, and upon association with different substrate surfaces. In addition to comparison of the fibril structure and behavior of MinE^1-31^ under varying conditions, the study also broadens our understanding of the versatile behavior of amyloid-substrate surface interactions.

## Introduction

Amyloid fibrils are fibrillar protein aggregates that are caused by protein misfolding and abnormal assembly. They play a crucial role in human diseases such as Alzheimer's disease, Parkinson's disease, and diabetes mellitus type 2 [1–3]. Functional amyloids, that are identified under physiological contexts, have also been identified within the past decade in bacteria, fungi and insects as well as in humans [4]. In addition, amyloid fibrils have been progressively gaining attention in nanotechnology and biomaterials applications because of their unique physical and mechanical properties [5]. Therefore, understanding the fibrillation processes is important because it can point the way to treatments for the diseases with amyloidosis or create new application possibilities for technological purposes.

Atomic force microscopy (AFM) is one of the few techniques that allow imaging biological materials in aqueous solutions with nanometer resolution. AFM has been employed to observe various morphologies of amyloid fibrils in their native conformation [6–8]. Another advantage of AFM is the potential application in recording the assembly process of biological macromolecules through time-lapse imaging. Such application in studying the self-assembly process of amyloid fibrils can provide crucial information for understanding the aggregation mechanism of peptides into amyloid fibrils, which is a fundamental but important issue in designing and controlling peptide assembly for practical applications [4,9–11]. However, observations of the fibril formation processes are rare so far [12].

Previous AFM studies of amyloid fibrils were mainly conducted with the tapping mode, the most widely used AFM technique [3,6–8,12,13]. However, the force sensitivity of the tapping mode is seriously reduced when operated in aqueous solutions due to the damping caused by the hydrodynamic interaction between the AFM cantilever and the liquid. Several recent studies have demonstrated that the frequency-modulation (FM) detection exhibits a superior force sensitivity in solutions compared with the tapping mode [14–16]. In this work, we studied the morphology and growth of the amyloid fibrils formed by MinE^1-31^ in solutions using frequency-modulation atomic force microscopy (FM-AFM). The technique enables applying a small force over the surface thereby preventing the soft and fragile biological samples from deformation or destruction. This is important, because many self-assembly structures tend to be fragile during the initial stage of formation. However, very few studies on amyloid fibrils have been conducted with FM-AFM due to the complexity in its operation. Fukuma et al. successfully imaged the molecular-level surface structures of islet amyloid polypeptide (IAPP) fibrils and *α*-synuclein protofibrils by using FM-AFM [17]. Here, we used FM-AFM to study the self-assembly of the N-terminal domain of MinE (MinE^1-31^) into amyloid fibrils on mica as well as on supported membranes under various buffer conditions. We showed that salt and sucrose affected fibril curves as well as separation distance and stacking behavior of protofibrils of MinE^1-31^ on mica under aqueous environment. Furthermore, fibril morphology and protofibril organization was altered upon association with the membrane without causing detectable damage to the membrane. Thus the study demonstrates characteristics of a new type of the amyloid-like fibril on the different substrate surfaces as well as the self-assembly behavior of MinE^1-31^ at nanometer scale for understanding the MinE function.

**Fig 1:**
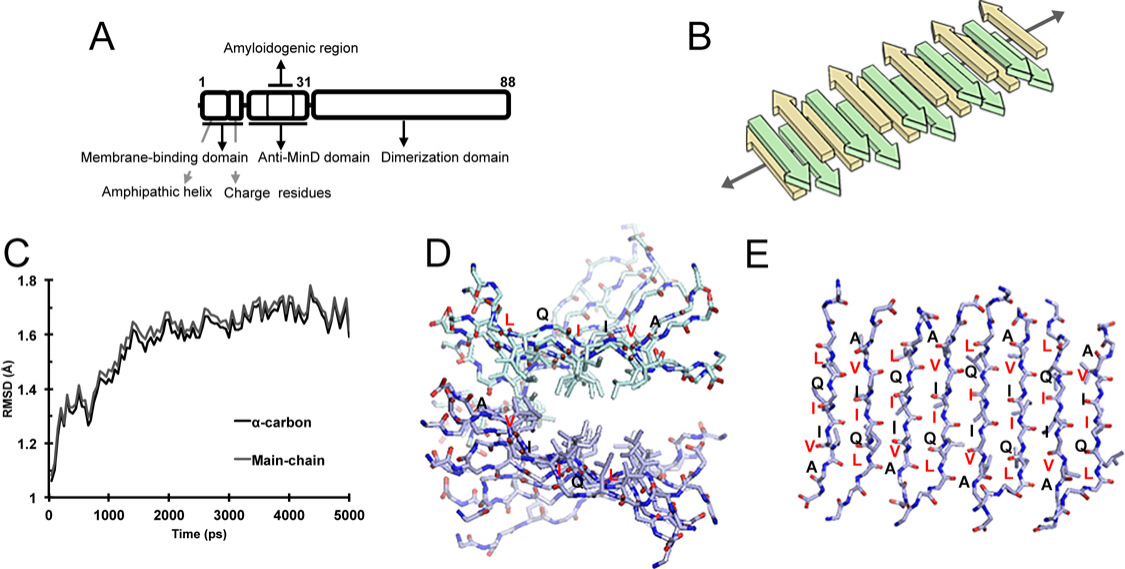
Organization of protein domains in MinE and model of the cross-β structure formed by the amyloidogenic region of MinE. **(A)** The MinE protein can be divided into three functional domains, a membrane-binding domain that contains a membrane-induced amphipathic helix and basic residues, a bifunctional domain that interacts with MinD in an α-helical conformation and self-assembles in a β-stranded conformation, and a dimerization domain at the C-terminus. The dimerization domain is also known as the topological specificity domain. **(B)** Illustration of the cross-β structure formed by the amyloidogenic region of MinE (19–28); the alternating β strands are colored green and yellow for clarity. **(C)** RMSD plots of α-carbon and main-chain atoms from a 5-ns simulation to demonstrate conformational equilibrium. **(D)** Frontal view of the cross-β structure of the amyloidogenic region of MinE^1-31^; only the backbone of the molecule and the side chains facing the hydrophobic interface are shown. **(E)** Top view of the model showing anti-parallel arrangements of the residues in the amyloidogenic region; residues containing side chains facing the hydrophobic interface of two β sheets are shown in red.

MinE is a component of the Min system that regulates the spatial precision of cell division sites in *E*. *coli* [18] and exhibits biochemical properties and self-assembly behavior reminiscent of those of typical amyloid fibrils [19]. MinE can be dissected into three functional regions: the N-terminal membrane-targeting motif (2–12), the MinD-interacting domain (13–31), and the C-terminal dimerization domain (32–88) (Fig 1A). It plays a critical role in triggering the oscillation of the Min proteins by forming a ring-like structure, the MinE ring, at the inner surface of the cell membrane, as has been demonstrated by fluorescence microscopy [20–22]. Recently, we reported that MinE self-assembles into fibrillar structures on the membrane that is mediated by its N-terminal domain (MinE^1-31^) [19]. However, the molecular basis of the formation of the MinE ring remains unclear. MinE^1-31^ carries a membrane-stabilized amphipathic helix and a cluster of positively charged residues to support interfacial interaction with the membrane [23–25] and an amyloidogenic fragment to mediate fibril formation [19] (Fig 1A and 1B). Fibril formation on lipid membrane is also found for both pathological and functional amyloid fibrils [3,23], but AFM studies remain rare [3,13,26,27]. The current work studied the behavior of protofibrils of MinE^1-31^ on a supported lipid bilayer and thus could provide valuable information for understanding the interplay between amyloid fibrils and biological membrane.

## Materials and Methods

### Molecular dynamics simulation

A molecular dynamics simulation was performed using Discovery Studio 3.5 (Accelrys Inc., San Diego, CA, USA). The starting model was constructed as described in the Results section, solvated, and a CHARMm27 forcefield was applied. The resulting molecule was simulated using the distance-dependent dielectric model and the standard cascade protocol consisted of two rounds of minimization, heating, equilibration, and production.

### Preparation of peptide solution and supported lipid bilayers

The MinE^1-31^ peptide was synthesized without modification, rehydrated in 20 mM Tris-Cl pH 7.5, clarified by centrifugation at 21,000 *g* for 30 min to remove precipitates, and quantified before preparation of the reaction mixtures [23]. The peptide solution was diluted and stored at 4°C for no more than a week before use. For examining fibril formation by AFM, the buffer was adjusted to imaging buffer A (20 mM Tris-Cl pH 7.5, 120 mM KCl, 100 mM sucrose), imaging buffer B (20 mM Tris-Cl pH 7.5, 150 mM KCl, 100 mM sucrose), or imaging buffer C (20 mM Tris-Cl pH 7.5, 150 mM KCl, 20 mM sucrose) prior to applying 25 μl peptide solution onto a freshly cleaved mica. It should be noted that, although the observed amount of the peptides adsorbed on mica varied from time to time due to some unknown factors, similar self-assembly behaviors and fibril morphology were consistently observed under the same buffer conditions.

The method of vesicle fusion used to prepare SLBs on mica is summarized below. 0.5 mg/ml *E*. *coli* polar lipids (Avanti) was vacuum dried and was rehydrated in a buffer containing 20 mM Tris-Cl pH 7.5, 200 mM sucrose. The liposome suspension was subjected to freeze-thaw cycles before passing through an extruder with filters of pore size 400 and 100 nm in sequential steps to generate small unilamellar vesicles (SUVs) [24]. The SUV suspension was diluted 10 fold with the same buffer and applied to a freshly cleaved piece of mica fixed on a round metal chip. The setup was incubated in a sealed chamber and heated on a hot plate at 50°C for 30 min followed by cooling at room temperature for another 30 min. To scan SLBs, additional 35 μl imaging buffer was applied to immerse the probe in solution. To scan the peptide-containing reactions, SLBs were first scanned to examine the planarity and surface coverage of the mica before addition of the peptide solution. The SLB buffer was replaced with 25 μl peptide solution and incubated at room temperature for 2 h before placing on the sample plate for AFM scanning [19].

### Frequency-modulation atomic force microscopy system

The FM-AFM imaging system was configured for an Agilent 5500 AFM (Agilent Technologies, Inc., Palo Alto, CA, USA) in which the bending of the cantilever was detected by measuring the deflection of a laser beam. The frequency modulation detection was enabled by a phase lock loop (PLL) control system (Nanosurf easyPLL plus system; Nanosurf AG, Liestal, Switzerland). We used stiff probes (Nanosensors PPP-RT-NCHR and PPP-NCHAuD; Nanosensors, Neuchatel, Switzerland) with a high force constant of ≃42 N/m and a high resonance frequency of ≃330 kHz in air (136.6 to 143.3 kHz in liquid). It should be noted that PPP-NCHAuD was coated with Au on its reverse side and therefore exhibited a high reflectivity for detecting the laser beam in water. All of the scans were performed under a constant amplitude (1.5 nm) and a constant frequency shift. The force applied to the sample was adjusted to strike a balance between image quality and minimizing damage to the samples. The scan speed was varied from 1500 to 3500 nm/s, depending on the size of the scanned area.

The MinE^1-31^ sample was adsorbed onto a freshly cleaved mica surface (6 mm in diameter) that was glued to a round metal chip, which was magnetically fixed onto the AFM stage. Before installation on the sample plate, additional imaging buffer (35 μl) was applied to the scanner nose to immerse the probe in solution. Both the scanner nose assembly and the AFM probe were cleaned with pure ethanol prior to scanning. After the sample was installed on the AFM stage, 30 min was required for the system to reach thermal equilibrium before commencing the AFM scans. We imaged three to six positions for each sample to ensure consistency among the observations. All of the scans were performed at room temperature in liquid.

### Transmission electron microscopy

MinE^1-31^ peptide (4 μl of 6 μM) prepared in three imaging buffers was spotted onto a Ultrathin Carbon Type A grid (Pelco®) and incubated for 1 min at room temperature. Prior to use, the grid was glow-discharged by the EMS 100 glow discharge unit for 15 seconds. The sample was then stained with 4% uranyl acetate for 70 sec followed by air drying. Transmission electron microscopy (TEM) images were collected using a Tecnai G2 F20 TWIN electron microscope with a field emission gun (FEI company) at 200 KeV, which was equipped with a Gatan UltraScan 4000 CCD Camera (4,096 × 4,096; 15-μm pixel size), and processed using Digital Micrograph software (Version 3.9.5, Gatan).

## Results

### Modeling the self-assembly core region of MinE^1-31^

To investigate the cross-β assembly underlying the fibril formation of MinE^1-31^, an oligomeric model was constructed based on a water-soluble cross-β structure of the peptide self-assembly mimic that was engineered from the *Borrelia* outer surface protein OspA [28,29]. The model demonstrated two β-sheets held together by hydrophobic interactions and each β-sheet contained eight strands arranged in an anti-parallel manner, which met the criteria of our proposed model of the self-assembled amyloidogenic region of MinE^1-31^ [19]. We therefore used the amyloidogenic fragment 19-KERLQIIVAE-28 of MinE^1-31^ to substitute for the repeating β-strand sequences in the cross-β structure of 3CKA (residues 118–209). The linkages between the repeating units were removed to separate the β-strands. The initial model was simulated for 5 ns, and the RMSD (root mean square deviation) plot indicated that the conformational trajectory had reached equilibrium (Fig 1C).

The resulting oligomeric model of the amyloidogenic region of MinE^1-31^ retained the characteristic cross-β architecture of amyloid fibrils (Fig 1D and 1E). The model demonstrated that the side chains of L22, I24 and V26 were oriented towards the hydrophobic interface between two β-sheets, and the side chains of R21 and Q23 were exposed on the surface (Fig 1D and 1E). In addition, a gradual twist and an asymmetrical shape along the long axis of the molecule were observed (Fig 1D). There were no aromatic residues in the amyloidogenic region; thus, the model exhibited no interdigitation of the side chains at the hydrophobic interface. The model was estimated to be approximately 2 nm wide and 2 nm thick near the center of the oligomer. This information was used as a reference for analyzing the packing order of the protofibrils into fibrils and bundles based on the AFM and TEM measurements described below.

### MinE^1-31^ self-assembles into protein fibrils on mica

In this work, we investigated the MinE^1-31^ fibrils mainly using FM-AFM to study the morphology and dynamic growth of the fibrils and the organization of the underlying protofibrils. The advantages of FM-AFM in force sensitivity and spatial resolution were clearly demonstrated by comparing the performance of the FM and tapping modes in imaging the same area of a sample using the same tip and the same oscillation amplitude under identical environmental conditions. S1 Fig shows that the FM mode could resolve the protofibrils of MinE^1-31^ (S1A and S1C Fig), whereas the tapping mode could not (S1D and S1F Fig). The corrugation height obtained using the tapping mode was also much smaller than that obtained using the FM mode (S1C and S1F Fig), demonstrating that the spatial resolution and force sensitivity of the FM mode were superior to those of the tapping mode. The deflection image acquired using the tapping mode showed some contrast related to the surface morphology, reflecting a stronger tip-sample interaction (S1E Fig). In contrast, there is little contrast in the deflection image acquired using the FM mode (S1B Fig). In addition, we estimated an applied force of 50–300 pN for FM-AFM from the resonance frequency shift versus the tip-sample separation and the feedback setpoint [30], which showed that a small deformation was induced in the samples. Based on above measurements, the FM mode applied a lower loading force on the sample, thereby causing less structural deformation to the sample.

**Fig 2:**
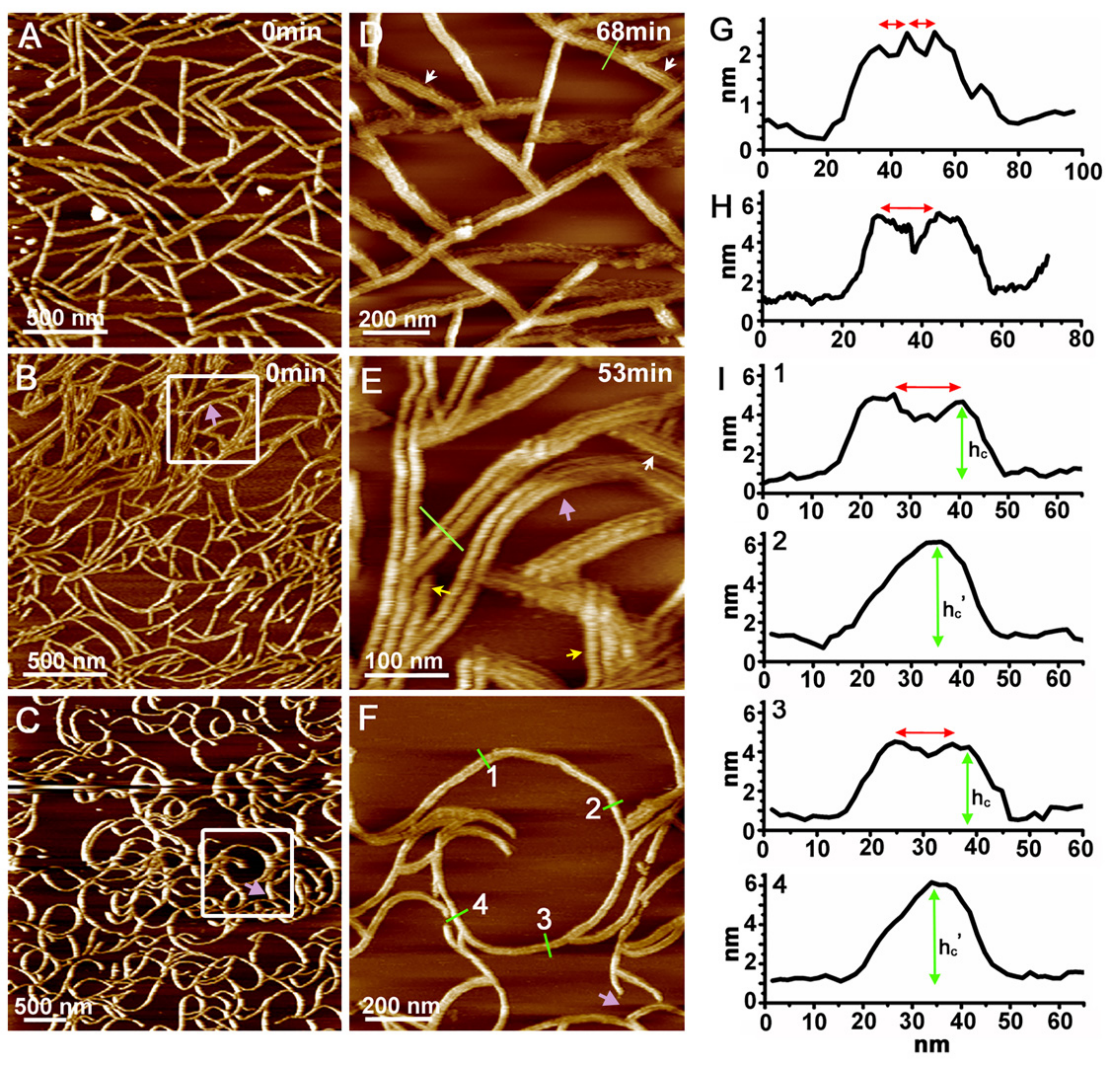
Self-assembly of MinE^1-31^ on mica under different buffer conditions. AFM images of MinE^1-31^ self-assembled into straight **(A** and **D)**, bent **(B** and **E)** and highly curved fibrils **(C** and **F)** in imaging buffers A, B and C, respectively. Graphs **(G–I)** show the height profiles corresponding to the green lines in images (D), (E) and (F). The four line profiles (I1–4) associated with (F) show alternating variations in the height (h_c_ = 3.5 ± 0.3 nm, *n* = 34; h_c_' = 4.7 ± 0.3 nm, *n* = 32; green double arrows) along the curved fibrillar structure. White arrows in (D) and (E) indicate where protofibrils are visible. (E) High resolution image of the outlined square region in (B). Purple arrow indicates newly growing fibril. It should be noted that the feature of double fibrils in (E) is not a result of the double-tip artifact, because triple fibrils are also observed, as indicated by yellow arrows. Red double arrows in (G, H, I1 and 3) indicate the separation distance between protofibrils (D) and fibrils (E and F). h_c_ and h_c_' denote the height of fibrils in imaging buffer C. The peptide concentration used in imaging buffers A, B and C, was 24, 41, and 18 μM, respectively.

The characteristics of the MinE^1-31^ fibrils were examined under three different conditions. In imaging buffer A (20 mM Tris-Cl pH 7.5, 120 mM KCl, 100 mM sucrose), straight fibrils that formed a web-like structure in areas with a high fibril density were observed (Fig 2A). The fibrils became noticeably curved at a higher salt concentration in imaging buffer B (20 mM Tris-Cl pH 7.5, 150 mM KCl, 100 mM sucrose; Fig 2B). Highly curved fibrils were observed at a lower sucrose concentration in imaging buffer C (20 mM Tris-Cl pH 7.5, 150 mM KCl, 20 mM sucrose; Fig 2C). The radius of curvature of the highly curved fibrils was approximately 203 ± 50 nm (*n* = 30). Taken together, the observations suggest that the lateral bending of the fibrils on a two-dimensional surface depends on the ionic strength of the environment and the effects of molecular crowding.

### Fibril morphology and packing order of protofibrils

**Fig 3:**
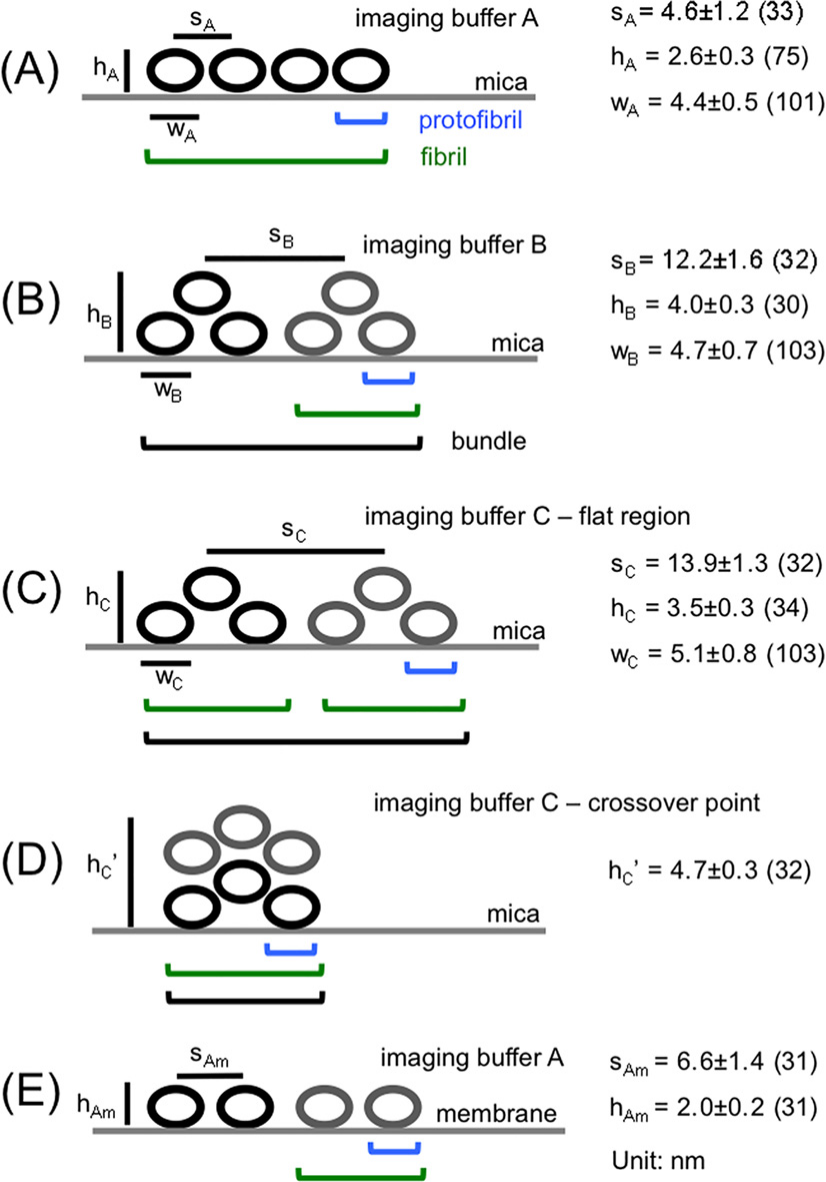
Packing order of the fibrillar structures formed by MinE^1-31^ under different conditions. Packing order of protofibrils adsorbed on mica and on a SLB in imaging buffer A shown in **(A)** and (**E),** respectively. Arrangement of fibrils in imaging buffers B and C are illustrated in **(B)**-(**D)**. The separation distance (s) between protofibrils or fibrils were obtained from AFM measurements of the distance between two adjacent peaks in the line profile of a bundle, neglecting measurement errors from the shape of the AFM probe tip. The height (h) of the fibrils was also measured by AFM, and the width (w) of the protofibrils was measured using TEM. Each circle represents the cross-section of a protofibril of MinE^1-31^. m denotes membrane.

Further details about the MinE^1-31^ fibrils were obtained from high-resolution images captured in selected regions. Most of the fibrils in imaging buffer A comprised 4–6 protofibrils that were arranged side-by-side with a lateral packing periodicity or a separation distance of 4.6 ± 1.2 nm (*n* = 33; Figs 2D and 2G and 3A). In contrast, a pair of fibrils with a separation distance of 12.2 ± 1.6 nm (*n* = 32) laterally associated to form bundles in imaging buffer B (Figs 2E and 2H and 3B). Arrays of protofibrils were only observed in a few regions, which may have resulted from the stacking of the protofibrils in imaging buffer B (Fig 3B). In addition, formation of new fibrils were observed (comparing Fig 2E with Fig 2B). The heights of the fibrils in imaging buffers A and B were measured to be 2.6 ± 0.3 nm (*n* = 75) and 4.0 ± 0.3 nm (*n* = 30), respectively (Fig 3A and 3B). These measurements showed that the fibrils observed in imaging buffer B likely consisted of two layers of protofibrils (Fig 3B), based on an oligomeric model of the amyloidogenic region of MinE^1-31^. However, for the highly curved bundles that were identified in imaging buffer C, an alternating height profile of the cross-section was observed along the curved bundle with a periodicity of approximately 450 nm (Fig 2F and 2I). This observation suggested that the fibrils were intertwined in a bundle, i.e., parallel fibrils were observed at locations corresponding to a height decrease, and the crossing points for the intertwined fibrils were observed at locations corresponding to a height increase (Fig 3C and 3D). The separation distance between parallel fibrils was determined to be 13.9 ± 1.3 nm (*n* = 32; Fig 2F and I-1 and I-3). In addition, the periodicity distance of the bundle twists appeared to be significantly larger than that of the fibril twists (82.0 ± 11.1 nm, *n* = 108) [19], suggesting that the structural complexity increased going from protofibrils to fibrils to bundles. The detailed features of the protofibrils could again not be resolved in most cases. However, there was no clear difference in the height profile to suggest that twists occurred along the bundles in imaging buffers A and B. It is likely that the adsorption force between the fibril and the mica surface was greater than the interfibrillar force, which prevented the fibrils from twisting.

**Fig 4:**
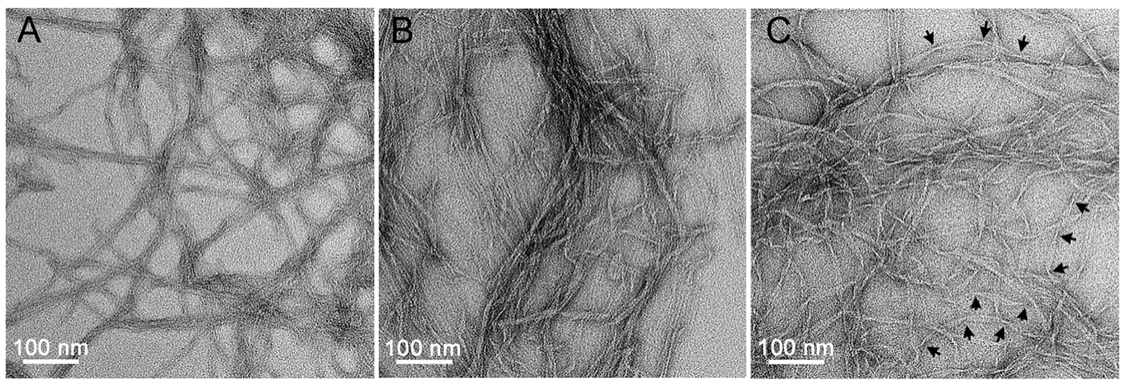
EM micrographs of MinE^1-31^ protofibrils and fibrils in imaging buffers A (A), B (B) and C (C). Arrows indicate the twisting points along the fibrils. The peptide concentration used for TEM imaging is 6 μM.

Unknown probe shapes and variations between different probes during AFM imaging may produce inaccuracies in width measurements; thus, we used negative-stain TEM to confirm the protofibril width (Fig 4). However, a technical drawback of using TEM was that the sample preparation procedure did not preserve the native morphology in solution due to steps of drying and staining. In addition, the imaging process was operated in vacuum. Thus, AFM and TEM served as complementary imaging techniques for our purposes of studying fibril morphology and estimating fibril dimensions. The widths obtained from the TEM measurements for the protofibrils that were prepared in buffers A, B and C were 4.4 ± 0.5 nm (*n* = 101), 4.7 ± 0.7 nm (*n* = 103) and 5.1 ± 0.8 nm (*n* = 103), respectively (Figs 3A–3C and 4A–4C). All of these measured widths were greater than the estimated width of the self-assembly core region, which may indicate that the N-terminal region preceding the amyloidogenic region affected the widths via an unknown mechanism. The width increase may simply due to a space occupation by the region preceding the amyloidogenic region. Alternatively, the substrate surface may have stabilized the β-strand conformation of the preceding region, leading to the observed width increase. These possibilities await to be further investigated.

### Dynamic assembly of MinE^1-31^ fibrils

**Fig 5:**
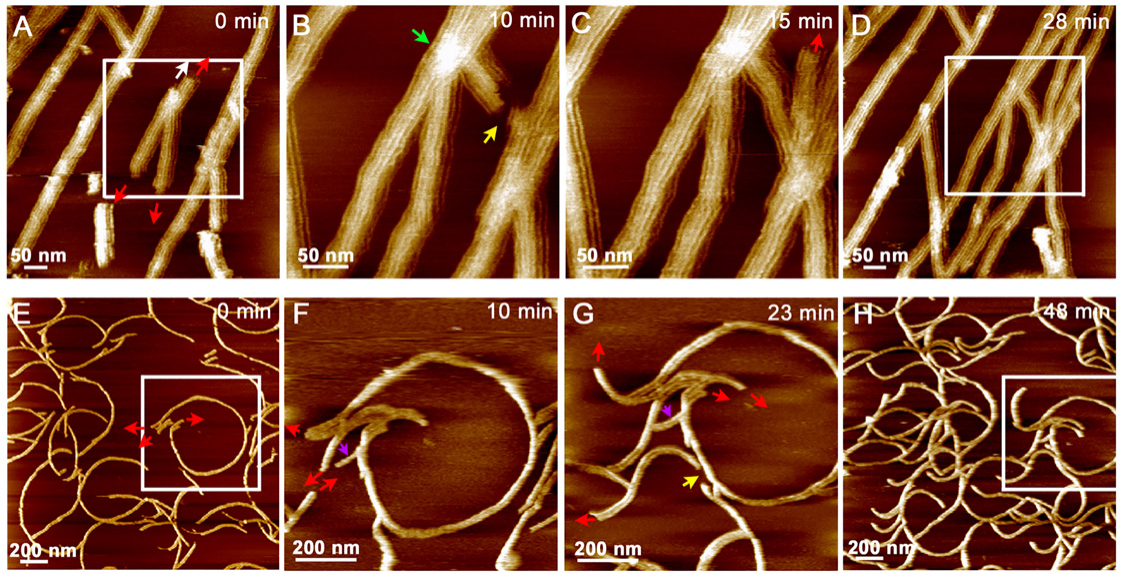
Growth of MinE^1-31^ fibrils on mica. **(A–D)** Elongation of straight fibrils in imaging buffer A. The peptide concentration was 12 μM. **(E–H)** Elongation of highly curved fibrils in imaging buffer C. The peptide concentration was 18 μM. The time interval in an image sequence depended on the scanning speed, which was determined by the instrument configuration and the size of the region of interest. Only selected images in each time sequence are shown. Red arrows indicate the direction of fibril elongation; yellow arrows indicate annealing between fibrils; white arrow indicates protofibrils growing along the existing fibrils; green arrow indicates a node-like region for branch formation. Occasionally we can see growth from single fibrils to paired fibrils. One is indicated by a purple arrow in (F) and (G).

The fibril growth was seen in many areas of the mica surface, revealing active assembly of MinE^1-31^ fibrils on the mica substrate surface immersed in solution (Fig 5). Several features of the assembly process are summarized below. First, growth occurred in isolated fibrils that were elongated bi-directionally (Fig 5A, 5E and 5G, red arrows). Second, annealing and fusion between fibrils occurred during growth (Fig 5B and 5G, yellow arrows). Third, new protofibrils were added to the side of an existing fibril, which increased the fibril width (Fig 5A, white arrow), suggesting that the original fibrils acted as a template for the assembly of additional protofibrils. There was no absolute uniformity in the width of the branches and the number of protofibrils in the growing branches. Fourth, the height of the node-like regions on the fibrils increased, indicating that excess peptides accumulated in the regions that subsequently became active regions for fibril elongation and branching of new fibrils (S2A and S2B Fig). Free peptides in solution may have also contributed to fibril growth. Fifth, the disassembly of short fibrils and static fibrils were also observed (S2C and S2D Fig). The former phenomenon suggested the active remodeling of the existing fibrils, especially at the early stages of fibril formation. The growth rate of fibrils is shown in S2C and S2D Fig. However, the rate of elongation varied with position because of multiple growth modes; thus, the fibril growth rate could not be clearly correlated with the protein concentration.

### Formation of MinE^1-31^ fibrils on SLBs

The function of MinE to drive pole-to-pole oscillation of MinD depends on membrane association [23,24]; thus, we studied how the membrane affected the self-assembly of MinE^1-31^ into fibrils. Before the addition of the peptide solution, we used FM-AFM to examine selected areas in each preparation of the SLBs to ensure that any subsequent changes on the surface of the SLBs were associated with MinE^1-31^ and did not result from pre-existing inhomogeneities in the bilayer. The thickness of a SLB that was prepared from *E*. *coli* polar lipids (phosphatidylethanoamine:phosphatidylglycerol: cardiolipin = 67:23:10%, w/w; Avanti Polar Lipids, Ltd., USA) was previously estimated to be 5.0 ± 0.4 nm [19]. We further compared the SLB images acquired with the FM mode and the tapping mode by comparing the bilayer thickness measured at the edges of SLBs (S3 Fig). The thickness of the bilayer was estimated as 5 nm with the FM mode (S3B Fig) and as 4 nm with the tapping mode (S3D Fig), respectively. The higher value obtained with the FM mode indicates less deformation of the SLB surface due to its higher force sensitivity, again demonstrating suitability of FM-AFM in imaging biological samples.

**Fig 6:**
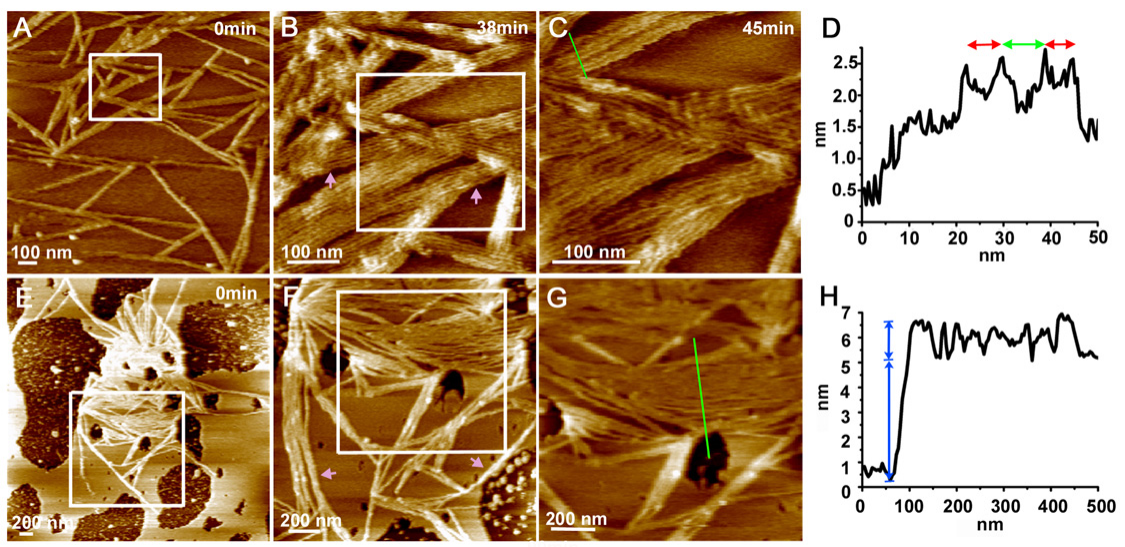
Self-assembly of MinE^1-31^ on SLBs. **(A–C)** Assembly of MinE^1-31^ on a high-coverage area of the SLBs. The peptide concentration was 24 μM. **(E–G)** Assembly of MinE^1-31^ on broken SLBs. The time for each set of data is indicated at the upper-right hand corner of the images. Notice that (G) was taken before (E) and (F). Newly assembled fibrils can be observed between (A) and (B) and between (E) and (F). The peptide concentration used was 12 μM. Graphs (B) and (C) and (F) and (G) are images of (A) and (B) at higher resolution, respectively. Graphs (D) and (H) show the height profiles corresponding to the green lines in images (C) and (G). Experiments were performed in imaging buffer A. Red double arrows indicate the separation distance between protofibrils. Green double arrow indicates the separation distance between fibrils. Purple arrows indicate the newly formed fibrils comparing to the previous images. Blue line indicates the thickness of supported lipid bilayer.

Clear differences in the self-assembly behaviors of MinE^1-31^ on the SLBs were observed (Fig 6A–6C and 6E–6G). Varying numbers of protofibrils were aligned in parallel into ribbons with a separation distance of 6.6 ± 1.4 nm (*n* = 31; Fig 6B, 6C, 6F and 6G), which was wider than the separation distance observed on mica. The wider separation distance of protofibrils on the membrane was likely mediated by the membrane-binding motifs at the N-terminal domain of MinE. However, two distinct separation distances between the protofibrils were apparent at a higher magnification (Fig 6C and 6D), suggesting that a pair of protofibrils associated laterally into a fibril that further associated into ribbons. The height of the protofibrils that were identified on the SLBs was estimated to be 2.0 ± 0.2 nm (*n* = 31) above the bilayer (Fig 6D and 6H), which suggested that the fibrils that formed on the SLBs were thinner than those formed on mica. The observed difference between the thickness of the fibrils on the SLBs and mica may be explained by partial insertion of the fibrils into the membrane. Alternatively, the difference may have resulted from the distinct properties of the substrate surfaces and a reciprocal protein-substrate surface interaction. Moreover, there were no distinct height changes in the SLBs near the MinE^1-31^ ribbons to indicate that the fibrils imposed any stress on the membrane. In areas with low membrane coverage, ribbons formed exclusively on the membrane (Fig 6E–6G), and only aggregates were observed on the bare mica surface, i.e., the broken regions of the SLBs, suggesting that MinE^1-31^ self-assembly was favored on the membrane. Note that growth dynamics showing newly formed fibrils were also observed for images acquired at different times. For example, images in Fig 6B and 6C were acquired 38 min and 45 min, respectively, after the image in Fig 6A. Fig 6F were acquired 90 min after that in Fig 6E.

## Discussion

In this study, we used a highly force-sensitive detection technique, FM-AFM, to characterize water-soluble fibrillar structures of MinE^1-31^ on mica as well as on lipid membrane. We observed that environmental factors such as ionic strength and molecular crowding determined the morphology and organization of the fibrils at different scales. These factors affected the curvature and coiling of the fibrils as well as the separation distance and the packing order of the underlying protofibrils. In these cases, salt may affect the folding stability and kinetics by changing the electrostatic repulsive force of a protein molecule [31]. Sucrose can function as a macromolecular crowding agent that stabilizes a labile protein, facilitates folding and refolding kinetics and enhances self-association [32]. In addition, sucrose may function as an osmolyte to alter the hydration state of a protein, thus promote protein compaction and folding [33], which further reflected on the self-assembly behavior. These factors can change the folding propensity of a peptide, the assembly of peptides into a protofibril, the lateral association of protofibrils into a fibril, and the interaction between fibrils. In addition, the same factors can also affect the protein-substrate interaction that further alters the fibril formation [34], which is reflected in the morphological changes of the fibrils on mica under different buffer conditions. The substrate effect was also demonstrated by the fibril on mica and on lipid membrane. The MinE^1-31^ fibrils appeared to form on the membrane rather than on mica (Fig 6E–6G), indicating a higher preference for the membrane interaction. The importance of substrate properties was further emphasized by the behavior of the MinE^1-31^ fibrils on the SLBs, which apparently altered the fibril morphology and protofibril organization. In addition, flexibility of the membrane may allow shallow insertion of the fibrils into the bilayer, resulting in a reduced thickness of the fibril above the membrane surface. All of the aforementioned interactions should eventually manifest at the fibril level. It should be noted that although different assembly kinetics and behavior of MinE^1-31^ may occur on SLBs, the formation of MinE^1-31^ fibrils is independent of membrane association.

The membrane is known to act as a catalyst to facilitate the formation of amyloid fibrils. For example, anionic phospholipids in the membrane can initiate the formation of amyloid fibrils from a range of proteins, including lysozyme, under physiological conditions [3,35,36]. Recruitment of aggregate-prone conformers to the membrane surface can increase the local protein concentration, which in turn can facilitate the assembly of amyloid fibrils [36]. Human islet amyloid polypeptide (hIAPP) is inserted into the negatively charged membrane through the N-terminal domain, which facilitates the formation of oligomeric intermediates. The membrane-bound oligomers subsequently misfold and mature into a β-stranded structure, resulting in fibrillogenesis. However, the mature hIAPP fibril does not associate with the membrane [37,38]. α-Synuclein interacts with the anionic surface of a membrane through the N-terminal amphipathic α-helix. This membrane association nucleates the protein aggregation of α-synuclein through a hydrophobic, non-Aβ component (NAC) adjacent to the N-terminal domain. Maturation of the aggregates transforms α-synuclein into a β-stranded conformation, resulting in amyloid-like fibrils [39–41]. These phenomena share common features of membrane interaction and fibril formation, irrespective of the pathology and origin of the proteins. Interestingly, similar behavior has been observed for the MinE-membrane interaction, including a preference for anionic phospholipids [23,42,43], a membrane-induced amphipathic helix at the N-terminus [24] and a region that can confer a cross-β structure for fibril formation. Thus, these studies suggest a tight link among structure-function relationships, protein-membrane interactions, and the formation of amyloid-like fibrils that could be widespread in nature.

Finally, membrane damage from folding intermediates or mature fibrils could contribute to the cytotoxicity of amyloid diseases. The oligomeric form of IAPP could cause membrane leakage via a pore-forming mechanism, similar to that of many antimicrobial peptides [44]. α-Synuclein causes damage to the membrane by lipid extraction and induces the formation of radiating fibrils from liposomes [45,46]. In addition, fragmented fibrils of β_2_-microglobin (β_2_m) have been shown to strongly interact with the membrane and extract small vesicles from liposomes [47]. Although MinE^1-31^ has previously been shown to induce the tubulation and collapse of liposomes [24], in this study, we found no evidence of membrane damage by the MinE^1-31^ fibrils on SLBs. However, in a previous study, we demonstrated that MinE and MinE^1-31^ induced clustering of fluorescent-phospholipid on SLBs [23], suggesting lipid redistribution occurred upon the formation of membrane-associated fibrils.

## Conclusions

A highly force-sensitive technique FM-AFM is used to characterize the amyloid-like fibrils formed by the N-terminal domain of MinE, MinE^1-31^. The fibrillar structures are characterized at the nanometer scale, allowing identification of the protofibril organization under environments of different ionic strength and molecular crowding. The protein fibrils undergo further changes on an artificial membrane surface without causing detectable damage to the membrane. An oligomeric model of MinE^1-31^ in a cross-β arrangement and the negative stain TEM are used to assist comprehension of the fibril organization and the differences between fibrils observed under different conditions. This study not only provides details of the MinE^1-31^ fibrils at the nanometer scale, but also broadens our understanding of the versatile behavior of amyloid-substrate interactions.

## Supporting Information

S1 Fig Comparison of AFM images of MinE^1-31^ fibrils acquired using FM and tapping modes. The resonance frequency of the cantilever was ~136.62 kHz, the free oscillation amplitude was ~1.5nm (3 nm peak-to-peak). **(A)** Height image acquired with the FM mode (△*f* = -68.86 Hz). **(B)** Deflection image acquired simultaneously with the FM mode in the same area shown in (A). **(C)** Height and deflection profiles along the green line in (A). **(D)** Height image of the same area taken with the tapping mode (amplitude setpoint ratio = 96.5%) under an identical condition. **(E)** Deflection image acquired simultaneously with the tapping mode in the same area in (D). **(F)** Height and deflection profiles along the green line in (D). Peptide concentration was 6 μM. The images were acquired in imaging buffer A.
(TIF)

S2 Fig Growing fibrils of MinE^1-31^ in imaging buffer A. **(A, B)** AFM image of a growing fibril (A) and height profile of the node-like region in the growing fibril (B). **(C, D)** Fibril elongation and fibril growth were analyzed. Micrographs (C) and (D) were captured 5 min 36 sec apart. Regions of interest (ROI) showing fibril growth are shown in the box in micrograph (D). The growth rate was measured at 1.4 nm/sec or 13.5 nm^2^/sec in ROI 1. Annealing of the growing fibril resulted in an overestimation of the growth rate above 0.2 nm/sec or 8.2 nm^2^/sec in ROI 2 and 0.3 nm/sec or 9.6 nm^2^/sec in ROI 3. A static fibril (red arrow) and disappearing aggregates (white arrows) were also observed. The peptide concentration was 12 μM.
(TIF)

S3 Fig AFM characterization of SLBs prepared on mica. **(A)** Topographic image of a low-coverage SLB on mica acquired with the FM mode in imaging buffer A. **(B)** Height profile along the green line in (A). The thickness of the bilayer was measured to be ~5 nm. **(C)** Topographic image of a high-coverage SLB on mica acquired with the FM mode in imaging buffer A. White arrows indicate the broken regions. The corrugation on surface of SLBs was measured as ~0.3 nm in (A) and (C). **(D)** Topographic image of a low-coverage SLB on mica acquired with the tapping mode AFM in imaging buffer A. **(E)** Height profile along the green line in (A). The thickness of the bilayer was measured to be ~4 nm, suggesting a deformation of SLB caused by the scan tip.
(TIF)
