# Supplementary figures and images for: Atomic Force Microscopy Characterization of Protein Fibrils Formed by the Amyloidogenic Region of the Bacterial Protein MinE on Mica and a Supported Lipid Bilayer
Source: PLoS One. 2015 Nov 12;10(11):e0142506. doi: 10.1371/journal.pone.0142506 (PMC4642933; doi:10.1371/journal.pone.0142506)

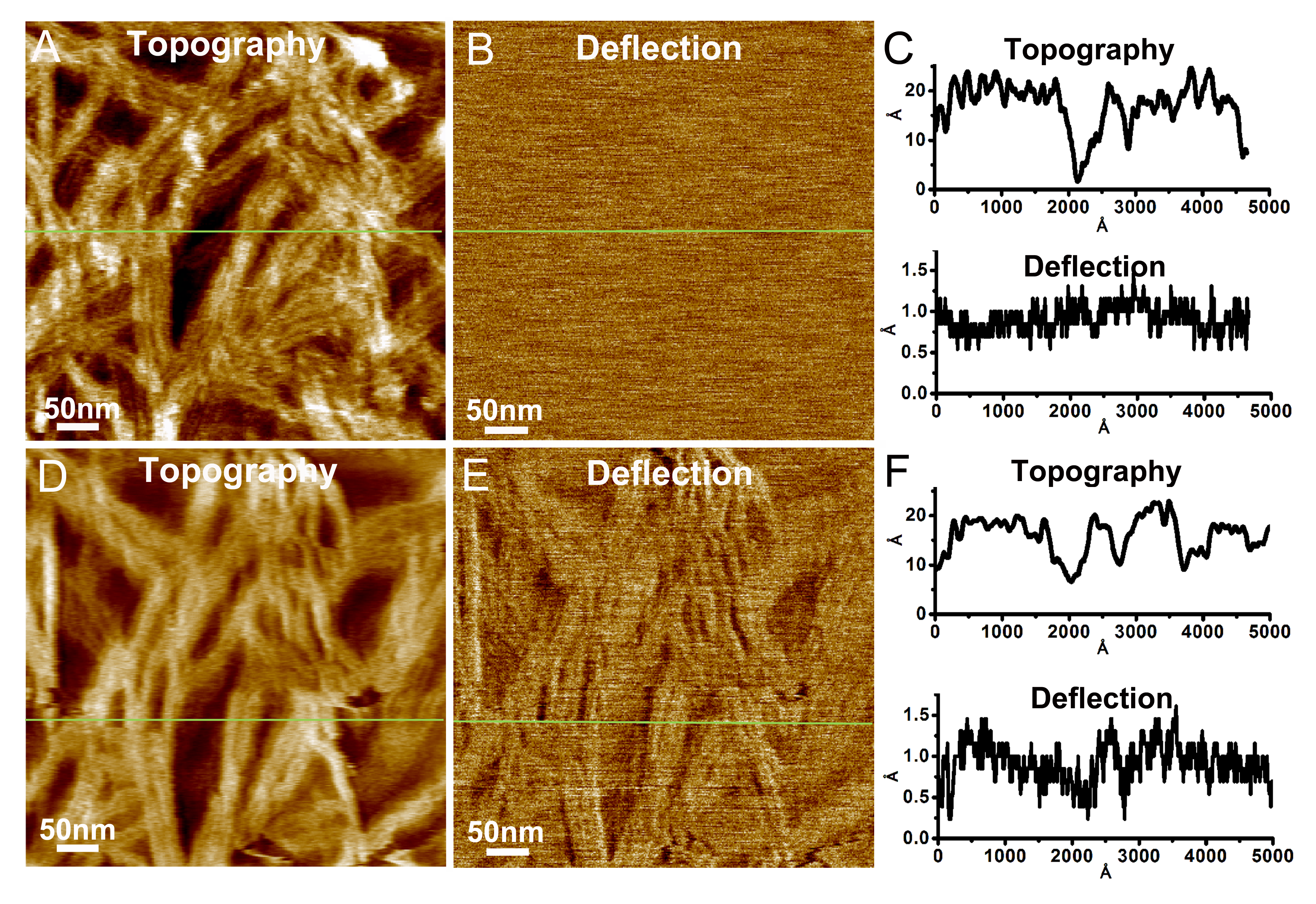

Supplement: S1 Fig — The resonance frequency of the cantilever was ~136.62 kHz, the free oscillation amplitude was ~1.5nm (3 nm peak-to-peak). (A) Height image acquired with the FM mode (△f = -68.86 Hz). (B) Deflection image acquired simultaneously with the FM mode in the same area shown in (A). (C) Height and deflection profiles along the green line in (A). (D) Height image of the same area taken with the tapping mode (amplitude setpoint ratio = 96.5%) under an identical condition. (E) Deflection image acquired simultaneously with the tapping mode in the same area in (D). (F) Height and deflection profiles along the green line in (D). Peptide concentration was 6 μM. The images were acquired in imaging buffer A. (TIF) [file pone.0142506.s001.tif]

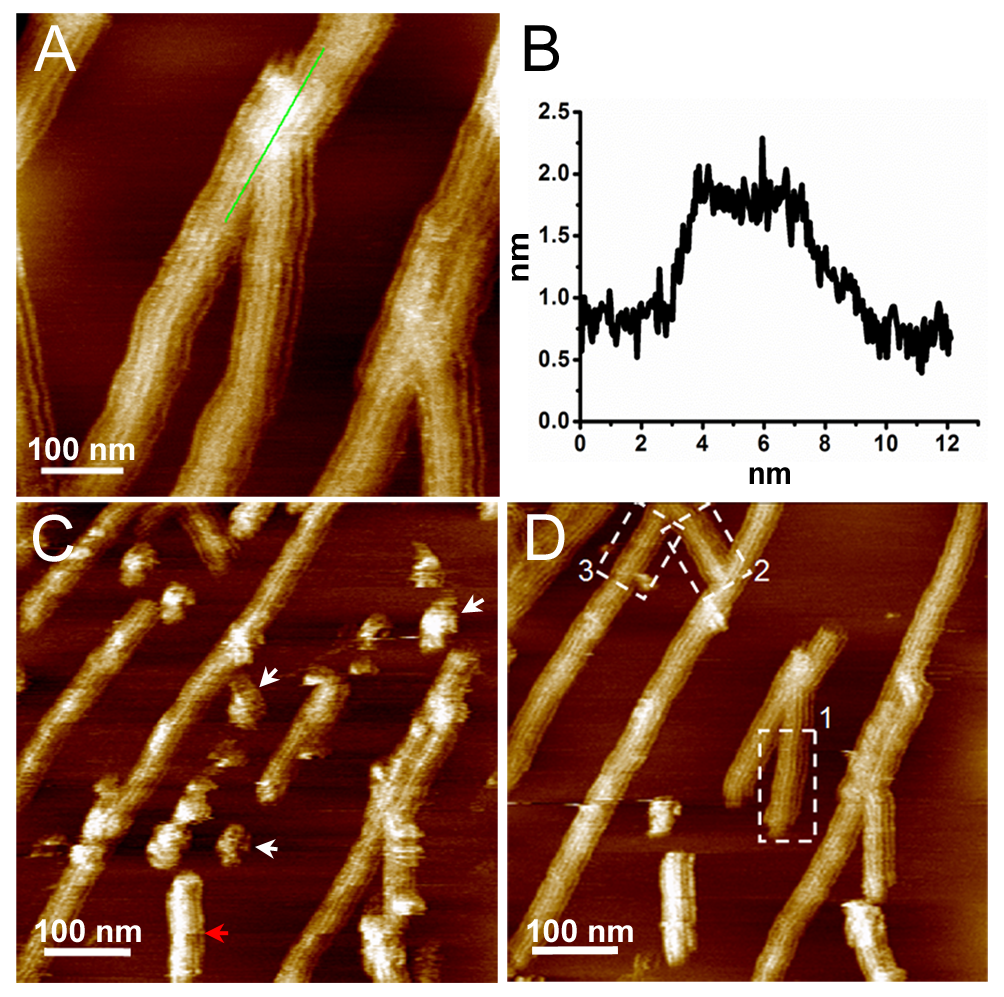

Supplement: S2 Fig — (A, B) AFM image of a growing fibril (A) and height profile of the node-like region in the growing fibril (B). (C, D) Fibril elongation and fibril growth were analyzed. Micrographs (C) and (D) were captured 5 min 36 sec apart. Regions of interest (ROI) showing fibril growth are shown in the box in micrograph (D). The growth rate was measured at 1.4 nm/sec or 13.5 nm2/sec in ROI 1. Annealing of the growing fibril resulted in an overestimation of the growth rate above 0.2 nm/sec or 8.2 nm2/sec in ROI 2 and 0.3 nm/sec or 9.6 nm2/sec in ROI 3. A static fibril (red arrow) and disappearing aggregates (white arrows) were also observed. The peptide concentration was 12 μM. (TIF) [file pone.0142506.s002.tif]

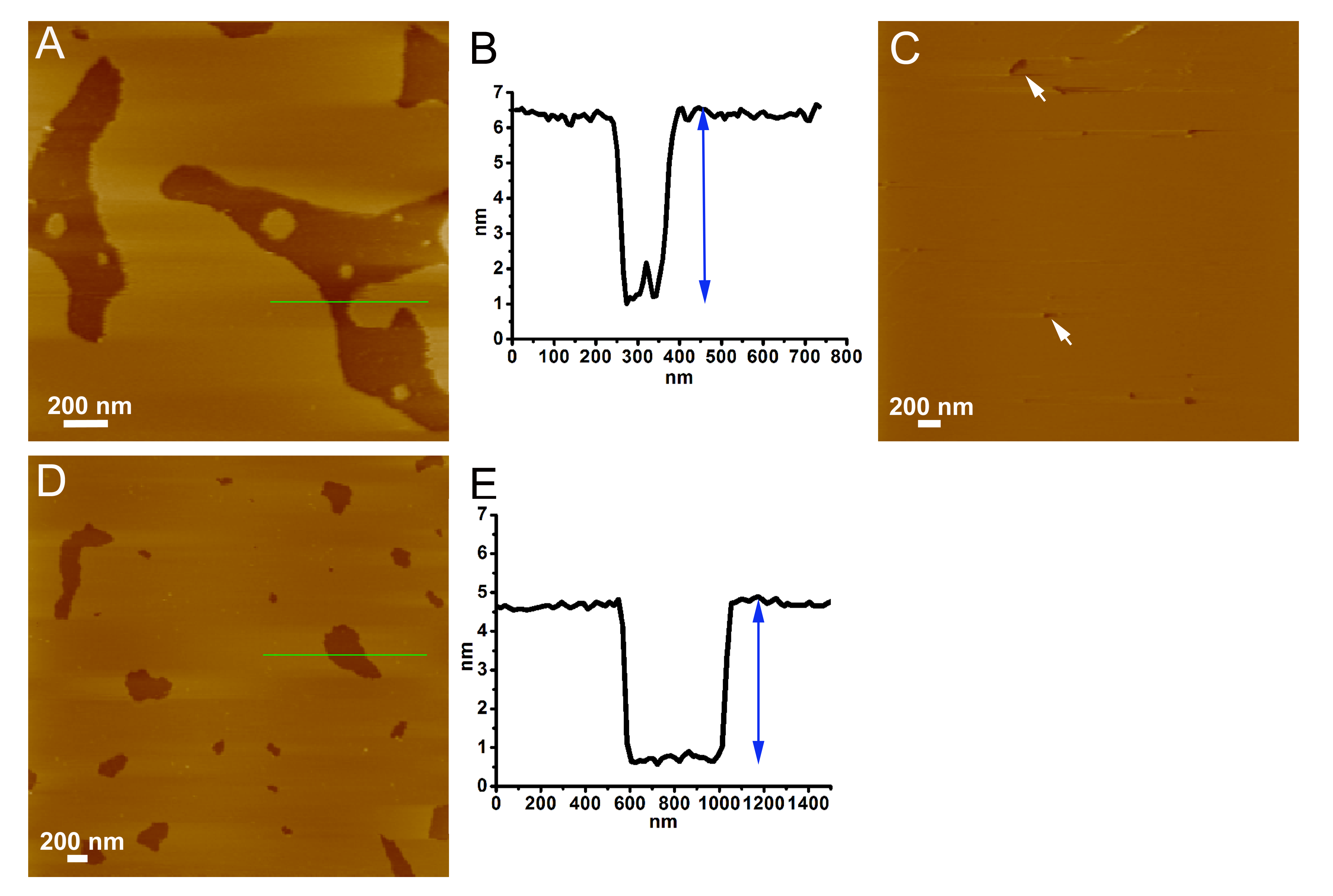

Supplement: S3 Fig — (A) Topographic image of a low-coverage SLB on mica acquired with the FM mode in imaging buffer A. (B) Height profile along the green line in (A). The thickness of the bilayer was measured to be ~5 nm. (C) Topographic image of a high-coverage SLB on mica acquired with the FM mode in imaging buffer A. White arrows indicate the broken regions. The corrugation on surface of SLBs was measured as ~0.3 nm in (A) and (C). (D) Topographic image of a low-coverage SLB on mica acquired with the tapping mode AFM in imaging buffer A. (E) Height profile along the green line in (A). The thickness of the bilayer was measured to be ~4 nm, suggesting a deformation of SLB caused by the scan tip. (TIF) [file pone.0142506.s003.tif]
